# Value of simplified lung lesions scoring systems to inform future codes for routine meat inspection in pigs

**DOI:** 10.1186/s40813-023-00324-y

**Published:** 2023-06-30

**Authors:** Joana Pessoa, Conor McAloon, Laura Boyle, Edgar García Manzanilla, Tomas Norton, Maria Rodrigues da Costa

**Affiliations:** 1 Pig Development Department, Teagasc Animal and Grassland Research and Innovation Centre, Moorepark, Ireland; 2 Section of Herd Health and Animal Husbandry, School of Veterinary Medicine, University College Dublin, Belfield, Ireland; 3 Present Address: Department of Reproduction, Obstetrics and Herd Health, Faculty of Veterinary Medicine, Ghent University, Salisburylaan 133, 9820 Merelbeke, Belgium; 4 M3-BIORES-Measure, Model and Manage Bioresponses, KU Leuven, Leuven, Belgium; 5 Present Address: Epidemiology Research Unit, Department of Veterinary and Animal Science, Northern Faculty, Scotland’s Rural College (SRUC), An Lòchran, 10 Inverness Campus, Inverness, IV2 5NA Scotland UK

**Keywords:** Abattoir, Benchmarking, Pig health, Pleurisy, Pneumonia

## Abstract

**Background:**

Across the European Union (EU), efforts are being made to achieve modernisation and harmonisation of meat inspection (MI) code systems. Lung lesions were prioritised as important animal based measures at slaughter, but existing standardized protocols are difficult to implement for routine MI. This study aimed to compare the informative value and feasibility of simplified lung lesion scoring systems to inform future codes for routine *post mortem* MI.

**Results:**

Data on lung lesions in finisher pigs were collected at slaughter targeting 83 Irish pig farms, with 201 batches assessed, comprising 31,655 pairs of lungs. Lungs were scored for cranioventral pulmonary consolidations (CVPC) and pleurisy lesions using detailed scoring systems, which were considered the gold standard. Using the data collected, scenarios for possible simplified scoring systems to record CVPC (n = 4) and pleurisy (n = 4) lesions were defined. The measurable outcomes were the prevalence and (if possible) severity scoring at batch level for CVPC and pleurisy. An arbitrary threshold was set to the upper quartile (i.e., the top 25% of batches with high prevalence/severity of CVPC or pleurisy, n = 50). Each pair of measurable outcomes was compared by calculating Spearman rank correlations and assessing if batches above the threshold for one measurable outcome were also above it for their pairwise comparison. All scenarios showed perfect agreement (k = 1) when compared among themselves and the gold standard for the prevalence of CVPC. The agreement among severity outcomes and the gold standard showed moderate to perfect agreement (k = [0.66, 1]). The changes in ranking were negligible for all measurable outcomes of pleurisy for scenarios 1, 2 and 3 when compared with the gold standard (rs ≥ 0.98), but these changes amounted to 50% for scenario 4.

**Conclusions:**

The best simplified CVPC scoring system is to simply count the number of lung lobes affected excluding the intermediate lobe, which provides the best trade-off between value of information and feasibility, by incorporating information on CVPC prevalence and severity. While for pleurisy evaluation, scenario 3 is recommended. This simplified scoring system provides information on the prevalence of cranial and moderate and severe dorsocaudal pleurisy. Further validation of the scoring systems at slaughter and by private veterinarians and farmers is needed.

## Background

The pig industry has long called for better feedback of meat inspection (MI) data to farmers [1]. Indeed, it is well accepted that MI can support animal disease control and identify and prosecute animal welfare issues [2]. Furthermore, the importance of MI as a valuable animal disease surveillance tool is recognized [3–7]. However, there is still no legal obligation for slaughterhouses to provide feedback on MI outcomes. Nonetheless, several European countries have standardized computer-based MI code systems to register findings during MI [8, 9]. The associated databases can assist farmers and their private veterinarians in disease surveillance, development of prevention strategies, and benchmarking [10–12]. Indeed, recent work highlights the need for a downstream (slaughterhouse-to-farm) exchange of information to achieve an integrated approach to the food chain [13].

However, due to the lack of standardization between slaughterhouses, and between official veterinarians (OVs) and official auxiliaries (OAs), several studies conclude that caution should be used when utilizing routinely collected MI data for other purposes than the protection of public health [4, 6, 11, 14]. Across the EU, efforts are being made to achieve modernisation and harmonisation of MI code systems, which are seen as beneficial to enable improvements in public health, and animal health and welfare [8].

Slaughterhouse checks, namely lung lesion scoring, are valuable to depict a farm’s respiratory health status [15, 16]. Several studies demonstrate the usefulness of MI data for respiratory disease surveillance [4, 12, 17]. Recently lung lesions were also prioritised as animal-based measures at slaughter for assessing the welfare of pigs on farm [9]. Indeed, there are several standardized protocols to record lung lesions [18–22]. Unfortunately, these are difficult to implement for routine MI tasks, as they are too detailed and time-consuming for OVs and OAs to register, when faced with ever increasing slaughter line speeds [23].

Respiratory disease has a major negative impact on the efficiency and sustainability of pig production worldwide. Therefore, it is urgent to develop tools that can aid the implementation of eradication and control strategies. Furthermore, routine information on lung lesions is a priority demand of stakeholders in the pig industry [24]. It is possible that lung lesion scoring systems could be simplified in order to collect information in a time period that facilitates normal slaughter line speed. Several options are possible for simplifying existing scoring systems, however it is not known how such modifications might impact on the accuracy of the modified examination procedure.

The aim of this study is to compare the informative value and feasibility of simplified lung lesion scoring systems to inform future codes for routine *post mortem* MI.

## Methods

### Data collection

Data on lung lesions were collected through visits to nine slaughterhouses (seven in the Republic of Ireland and two in Northern Ireland, United Kingdom) from December 2016 to May 2018, targeting 83 Irish pig farms. At least one batch per farm was assessed. A batch was defined as all pigs from one farm sent for slaughter on the same day.

### Lung lesion scoring at slaughter

All lungs were examined by the same veterinarian. Lungs were scored for cranioventral pulmonary consolidation and pleurisy lesions. CVPC was assessed using the scoring method developed by Madec and Derrien [20]. Each lung lobe was individually scored (each pair of lungs has seven lobes, namely the right and left apical, right and left cardiac, right and left diaphragmatic, and the intermediate lobe). The scores were 0 (no CVPC), 1 (1–25% of the lung lobe affected), 2 (26–50%), 3 (51–75%) and 4 (76–100%). The overall lung surface affected was also estimated and it accounted for lobe weights, as per Christensen et al. [18]. Briefly, the percentage of each lobe’s affected area was multiplied by the lobe’s relative weight and summed to provide the total weight percentage of affected lung.

Dorsocaudal pleurisy was scored using a modified version of the Slaughterhouse Pleurisy Evaluation System (SPES), developed by Dottori et al. [25]. The scores were 0 (no pleurisy), 2 (focal lesions in one diaphragmatic lobe), 3 (bilateral adhesions or monolateral adhesions affecting more than 1/3 of the diaphragmatic lobe), and 4 (extensive lesions affecting more than 1/3 of both diaphragmatic lobes). Cranial pleurisy, which refers to adhesions between the surface of the apical and cardiac lobes, and/or adhesions between the lung and the heart, was either absent or present.

These detailed CVPC and pleurisy lesions scoring systems were considered the gold standard.

### Simplified lung lesion scoring systems

Using the data collected, different scenarios for possible simplified lung lesion scoring systems to record CVPC and pleurisy lesions were defined. Prevalence and (if possible) severity scoring were calculated by transforming the data described above. When simplifying the scoring systems, we had two concerns in mind: 1) the value of information generated and 2) their feasibility under normal MI procedures.

Table 1 shows the detailed description of each scenario to assess CVPC. Scenario two was developed by Steinmann et al. [22] and it is currently used to score CVPC in all slaughterhouses across Germany [12].

**Table 1:** Summary of the lung scoring systems for the evaluation of cranioventral pulmonary consolidation (CVPC) at batch level

Scenario ID	Description of the scoring system	Measurable outcome at batch level			
		Prevalence	% of affected lung surface	N lobes affected	
Gold standard	Detailed description in section”Lung lesion scoring at slaughter”	Yes	Yes	Yes	
Scenario 1 Presence or absence of CVPC	CVPC was scored as present [1] or absent (0) for each pair of lungs	Yes	No	No	
Scenario 2 German scoring system	CVPC was scored as mild (lesions affecting < 10% of the lungs surface), moderate (10–30%), and severe (> 30%)	Yes	Yes	No	
Scenario 3 Number of lung lobes affected	CVPC was assessed in each lung lobe. The number of lung lobes affected was recorded (0–7)	Yes	Yes^a^	Yes	
Scenario 4 Number of lung lobes affected excluding the intermediate lobe	Pneumonia lesions were assessed in both apical, cardiac and diaphragmatic lobes. The number of lung lobes affected was recorded (0–6)	Yes	Yes^b^	Yes	

^a^% of lung surface affected was calculated attributing the same lobe weight to all lobes (n = 7)

^b^% of lung surface affected was calculated attributing the same lobe weight to all lobes (n = 6)

Table 2 shows the detailed description of each scenario to assess pleurisy lesions.

**Table 2:** Summary of the lung scoring systems for the evaluation of pleurisy lesions at batch level

Scenario ID	Description of the scoring system	Measurable outcome at batch level			
		Prevalence of CP^a^	Prevalence of DC^b^	Prevalence of Pleurisy	
Gold standard	Detailed description in section “Lung lesion scoring at slaughter”	Yes	Yes	Yes	
Scenario 1 Presence or absence of CP or DC	Both CP and DC were scored as present (1) or absent (0) for each pair of lungs	Yes	Yes	Yes	
Scenario 2 Presence or absence of pleurisy (both CP or DC)	Pleurisy was scored as present (1) or absent (0) for each pair of lungs, independent of the lung region affected	No	No	Yes	
Scenario 3 Presence or absence of CP and moderate and severe DC	CP was scored as present (1) or absent (0). Only moderate (SPES^c^ score 3) and severe (SPES score 4) were considered	Yes	Yes	Yes	
Scenario 4 Retained lungs in carcass	Pleurisy was scored as present (1) or absent (0) when a pair of lungs (or part of them) was retained in the carcass due to pleural adhesions to the thoracic wall	No	No	Yes	

^a^Cranial pleurisy

^b^Dorsocaudal pleurisy

^c^Slaughterhouse Pleurisy Evaluation System

### Statistical analysis

R version 4.0.2 (R Core Team, Vienna, Austria) was used for the statistical analyses [26].

To compare the agreement between the measurable outcomes from each lung lesion scoring system, the methodology described by O’Neill et al. [27] to benchmark antimicrobial use using different indicators was utilized.

In short, the relationship between each pair of measurable outcomes for CVPC and pleurisy was assessed using Spearman rank correlations. An arbitrary threshold to define high prevalence and/or severity of CVPC and pleurisy was set to the upper quartile (n = 50 batches) for each measurable outcome.

Batches above this threshold were categorized as belonging to the “action zone”, whereby they could theoretically be targeted for the implementation of interventions to reduce the prevalence and/or severity of CVPC and pleurisy. Kappa coefficients were calculated for each pair of measurable outcomes to assess the overall agreement between benchmarking classifications (i.e., in action zone or not). The kappa coefficient measures the agreement between two rating methods and typically ranges from 1 (perfect agreement) to 0 which represents an agreement rate arising by random chance[28]. Negative values up to -1 are possible but extremely rare and they indicate an agreement “worse than expected” [29]. Lastly, for each pairwise comparison the change in rank for every batch between two measurable outcomes was calculated.

## Results

A total of 201 batches were assessed at slaughter, comprising 31,655 pairs of lungs. On average, each slaughterhouse, farm, and batch had 3,517 ± 3,480, 381 ± 239, and 158 ± 59 plucks assessed, respectively (range 129–10,293, 41–1,154, and 26–330, respectively).

The results for the measurable outcomes using the different lung scoring system for the evaluation of CVPC are presented in Table 3.

**Table 3:** Measurable outcomes for the lung scoring systems for the evaluation of cranioventral pulmonary consolidation (CVPC) using the scoring method developed by Madec and Derrien ([20]; gold standard) and the four simplified scenarios

Scenario ID	Measurable outcome					
	Prevalence (%)		% of affected lung surface		N lobes affected	
	Mean (± SD)	Median (min.–max.)	Mean (± SD)	Median (min.–max.)	Mean (± SD)	Median (min.–max.)
Gold standard	17 (± 1.5)	13 (0–65)	6.6 (± 2.75)	6.2 (1.25–17.30)	1.7 (± 0.48)	1.6 (1.00–3.2)
Scenario 1 Presence or absence of CVPC	17 (± 1.5)	13 (0–65)	Na^a^	Na	Na	Na
Scenario 2 German scoring system	17 (± 1.5)	13 (0–65)	8.5 (± 3.23)	8.0 (5.00–23.00)	Na	Na
Scenario 3 Number of lung lobes affected	17 (± 1.5)	13 (0–65)	24.0^b^ (± 6.80)	23.5 (14.29–45.71)	1.7 (± 0.48)	1.6 (1.00–3.20)
Scenario 4 Number of lung lobes affected excluding the intermediate lobe	17 (± 1.5)	12 (0–65)	28.0^c^ (± 7.83)	27.4 (16.67–53.33)	1.7 (± 0.47)	1.7 (1.00–3.20)

^a^Not applicable

^b^% of lung surface affected was calculated attributing the same lobe weight to all lobes (1/7)

^c^% of lung surface affected was calculated attributing the same lobe weight to all lobes (1/6)

The prevalence of CVPC at batch level was 17 ± 1.5% for both the gold standard and the four simplified scenarios. Scenario 4 had slightly different median values, which are due to the exclusion of the intermediate lobe. For scenario 1 the measurable outcomes that assess the severity of CVPC were absent, making it the least informative one. Using the German scoring system (scenario 2) led to a slight increase in the percentage of affected lung surface. In contrast, when using scenarios 3 and 4, the increase was marked, with an increase in the percentage of affected lung surface approximately 4 times higher. Nevertheless, when using the number of lung lobes affected by CVPC as a proxy to express lesion severity, scenarios 3 and 4 were identical to the gold standard.

The results for the measurable outcomes using the different lung scoring system for the evaluation of pleurisy lesions are presented in Table 4.

**Table 4:** Measurable outcomes for the lung scoring systems for the evaluation of dorsocaudal pleurisy using a modified version of the scoring method developed by Dottori et al. [25]; Slaughterhouse Pleurisy Evaluation System) and the presence or absence of cranial pleurisy (gold standard) and the four simplified scenarios

Scenario ID	Measurable outcome					
	Prevalence CP^a^ (%)		Prevalence DC^b^ (%)		Prevalence pleurisy (%)	
	Mean (± SD)	Median (min.–max.)	Mean (± SD)	Median (min.–max.)	Mean (± SD)	Median (min.–max.)
Gold standard	18.6 (± 15.59)	13.1 (0–70.4)	15.6 (± 16.64)	8.9 (0–82.4)	25.1 (± 21.74)	16.8 (0–91.18)
Scenario 1 Presence or absence of CP or DC	18.6 (± 15.59)	13.1 (0–70.4)	15.6 (± 16.64)	8.9 (0–82.4)	25.1 (± 21.74)	16.8 (0–91.18)
Scenario 2 Presence or absence of pleurisy	Na	Na	Na	Na	25.1 (± 21.74)	16.8 (0–91.18)
Scenario 3 Presence or absence of CP and moderate and severe DC	18.6 (± 15.59)	13.1 (0–70.4)	12.7 (± 13.82)	7.4 (0–70.59)	23.6 (± 20.47)	15.6 (0–88.24)
Scenario 4 Retained lungs in carcass	Na	Na	Na	Na	1.4 (± 3.74)	0 (0–43.43)

^a^Cranial pleurisy

^b^Dorsocaudal pleurisy

The prevalence of dorsocaudal pleurisy at batch level was 16 ± 16.6% for the gold standard and scenario 1. Scenario 3 only considered cases of moderate to severe DC, and therefore its prevalence was lower (12 ± 13.8%). The prevalence of CP was 19 ± 15.6% for the gold standard and scenarios 1 and 3. For scenarios 2 and 4 we could only estimate the prevalence of overall pleurisy, which was markedly lower when looking only at scenario 4, with 1.4 ± 3.74% of lungs with pleurisy. Scenario 4 only captures pleurisy cases that lead to adhesions to the thoracic wall, thereby representing the most severe cases of this lesion.

**Fig. 1:**
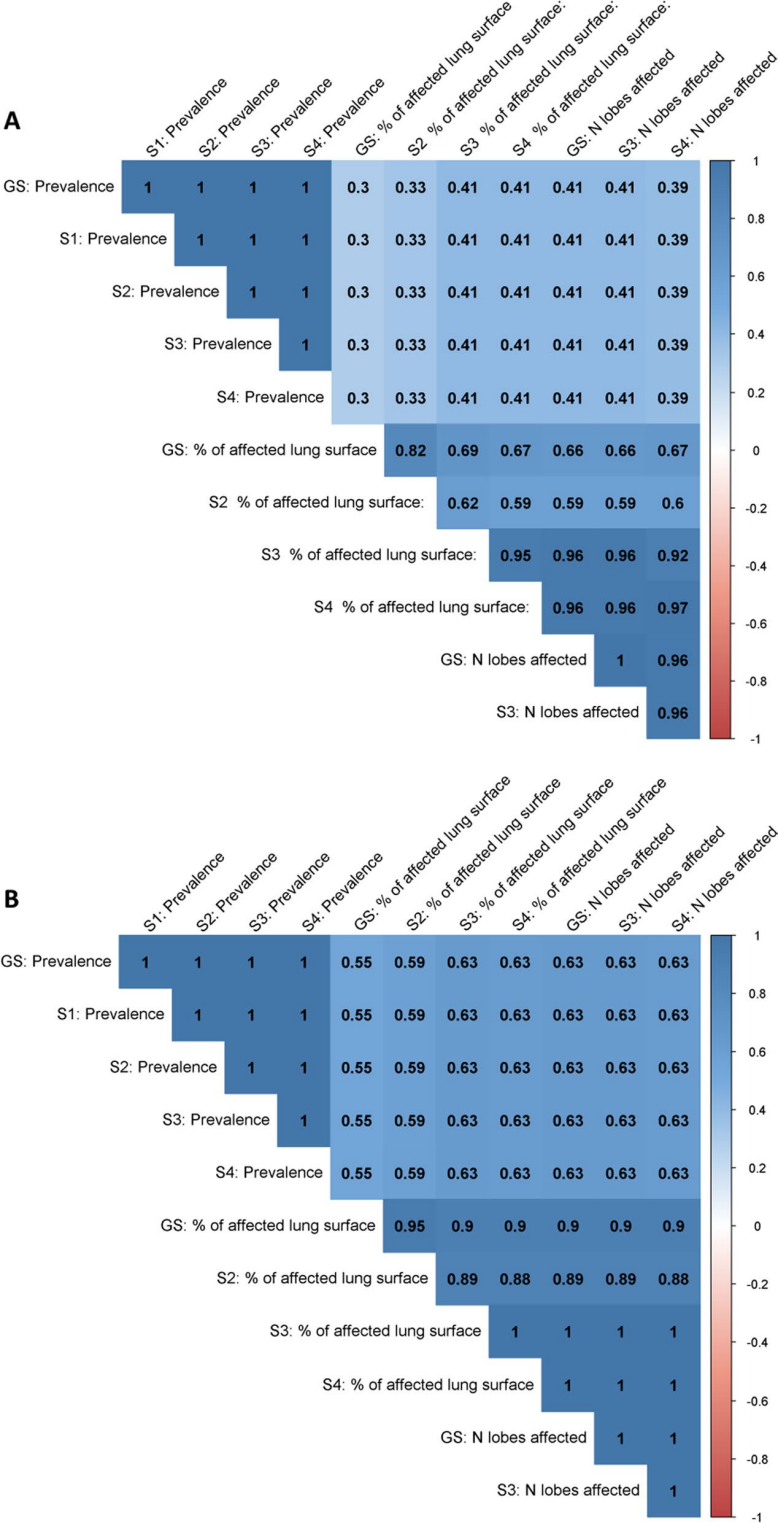
Pairwise comparison of measurable outcomes (Prevalence, percentage (%) of affected lung surface, and number (N) of lobes affected) for lung scoring systems for the evaluation of cranioventral pulmonary consolidation; 201 batches. The color of the tiles indicates the Spearman’s rank correlation coefficient of each pair of measurable outcome. **A** The values within the tiles indicate the kappa coefficient showing the agreement between the different outcomes. **B** The values within the tiles indicate the percentage of batches whose rank changed when comparing a given pair of measurable outcomes Legend: GS: gold standard; S 1-4: scenario 1 to 4.

Figure 1 summarizes the agreement between each pairwise comparison between the gold standard and the four scenarios for the evaluation of CVPC (1A); and the changes in ranking between each pair (1B).

When looking at the prevalence of CVPC, all scenarios showed perfect (k = 1) agreement when compared between themselves and with the gold standard (Fig. 1A). In contrast, when looking at the measurable outcomes related to the severity of CVPC (i.e., percentage of affected lung surface and number of lobes affected) there is low agreement (k = [0.30, 0.41]), indicating the presence of batches where there is high prevalence but low severity of CVPC and vice-versa. Still, when looking at the agreement among severity outcomes the gold standard shows moderate to perfect agreement (k = [0.66, 1]; Fig. 1A).

Regarding the percentage of batches whose rank changed when comparing a given pair of measurable outcomes (Fig. 1B), there were no changes when looking at prevalence of CVPC. However, the ranking changed when comparing prevalence and severity outcomes, but these changes were only moderate (55 to 63%), with the number of affected lobes (scenarios 3 and 4) showing the best relationship with the gold standard for prevalence of CVPC. When comparing the rankings between severity outcomes the relationship was strong (rs = 0.90–0.95).

**Fig. 2:**
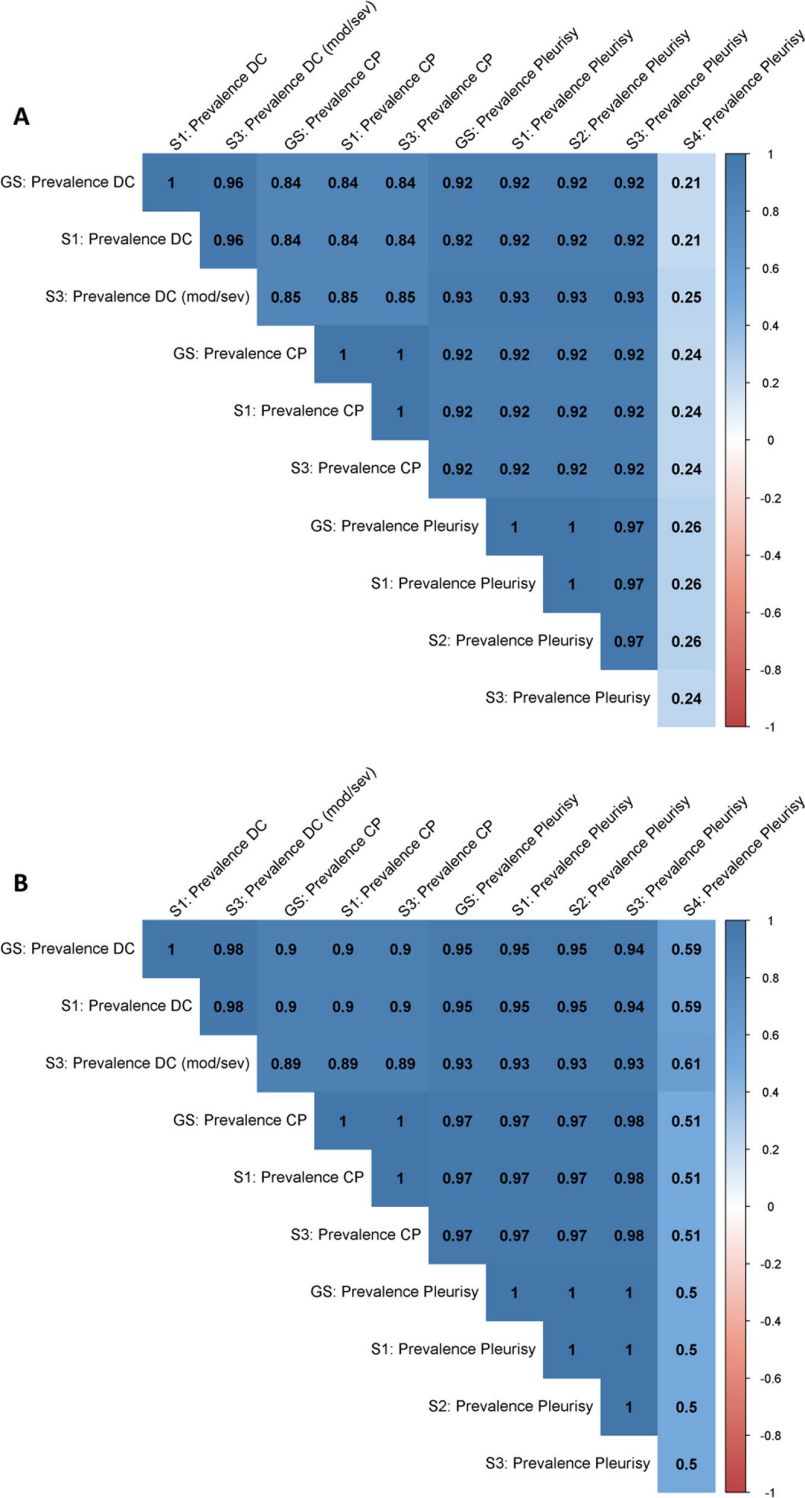
Pairwise comparison of measurable outcomes (Prevalence of dorsocaudal (DC), cranial (CP), and overall pleurisy) for lung scoring systems for the evaluation of pleurisy; 201 batches. The color of the tiles indicates the Spearman’s rank correlation coefficient of each pair of measurable outcome. **A** The values within the tiles indicate the kappa coefficient showing the agreement between the different outcomes. **B** The values within the tiles indicate the percentage of batches whose rank changed when comparing a given pair of measurable outcomes. Legend: GS: gold standard; S 1–4: scenario 1 to 4; mod/sev: moderate to severe

Figure 2 summarizes the agreement between each pairwise comparison between the gold standard and the four scenarios for the evaluation of pleurisy (2A); and the changes in ranking between each pair (2B).

The agreement between the gold standard for DC and scenarios 1 and 3 was perfect (k = 1) and 0.96, respectively. For CP, the agreement was perfect (k = 1). While for overall pleurisy, the agreement varied between perfect (for scenarios 1 and 2) to low for scenario 4 (k = 0.26).

Although the changes in ranking (Fig. 1B) were negligible for all measurable outcomes of pleurisy for scenarios 1, 2 and 3 when compared with the gold standard (rs ≥ 0.98), these changes amounted to 50% for scenario 4.

## Discussion

This study explored the effect of using simplified lung lesion scoring systems for the evaluation of CVPC and pleurisy lesions for routine meat inspection. Our approach highlighted the agreement between different scoring systems and a gold standard, and also how the scenarios considered can provide different degrees of information.

When simplifying the scoring systems, we had two concerns in mind: (1) the value of information generated to inform farmers, food business operators, and the competent authorities and (2) their feasibility under normal MI procedures, especially when slaughter line speeds are fast or human resources are low.

### Value of information

For farmers, data on pig respiratory health is a high priority [24] and collection of such data at slaughter is an easy, cheap and stress-free way of gathering information [30]. This information is useful to monitor the efficacy of disease control measures such as vaccination and treatment practices [23].

Findings from a recent Irish cross sectional study suggest that the effect of the generalized use of *Mycoplasma hyopneumoniae* vaccination in piglets led to the presence of mild CVPC, but not to a decrease in their prevalence [15]. This indicates the need for inclusion of severity outcomes when assessing CVPC at slaughter. Indeed, our findings show the presence of batches where there is high prevalence but low severity of CVPC and vice-versa. When looking at the different scenarios for the evaluation of CVPC suggested in this study, only scenarios 2–4 allow for the inclusion of severity scores (Table 1). Of these, scenario 2 shows the highest agreement when compared to the gold standard (k = 0.82). This scenario was included because it is already routinely used in German slaughterhouses [12]. Nevertheless, a recent study suggests that this scoring system is poorly reproducible [8]. Indeed, the interpretation of the percentage of lung affected with CVPC is subjective so we can expect a degree of bias between inspectors. Such bias can reduce farmer trust in the findings of the MI process [1].

Pleurisy is commonly identified as affecting either the cranial or dorsocaudal regions of the lungs to distinguish between different pathogens [23]. Dorsocaudal pleurisy is generally attributed to *Actinobacilus Pleuropneumoniae* [31]. While cranial pleurisy can be attributed to *Mycoplasma hyopneumoniae* infections [32, 33]. In our study, only scenarios 1 and 3 differentiate between cranial and dorsocaudal pleurisy, thus facilitating the process to achieve a presumptive diagnosis (Table 2). Scenario 3 could lead to an underestimation of the prevalence of dorsocaudal pleurisy as it only records moderate/severe cases. Nevertheless, there were only negligible differences in agreement (kappa = 0.98) when compared to the gold standard.

Benchmarking is useful to help farmers understand where they are positioned in comparison to their peers. Moreover, it also allows farmers to benchmark their farm over time. Our results show that different lung scoring systems may diverge when used to benchmark farms (Figs. 1B and 2B). The differences in ranking between scenarios were not substantial for pleurisy scoring, with the exception of scenario 4. However, when looking at the exchange between prevalence and severity scoring for CVPC, the differences were substantial (rs ≤ 0.63). Ideally, scenarios 2, 3, and 4 are preferable, as they provide information on both prevalence and severity of CVPC.

For food business operators, the generation of information that leads to lower costs and greater effectiveness is of the utmost importance [13]. The requirement for trimming or condemnation (partial or total) of lesions reduces the speed of the slaughter line [34]. This equates to inefficiencies and higher costs from the food business operators point of view. Scenario 4 for the evaluation of pleurisy registers only those lungs that lead to additional carcass trimmings. This is the most relevant scenario for these stakeholders.

For competent authorities data on lung lesions can aid in the design of national or regional control plans for specific pathogens [15] or relevant targeted actions to improve animal health and welfare [8, 9]. Although the lesions assessed in this study are not pathognomonic, they may indicate the presence of relevant pathogens such as *Mycoplasma hyopneumoniae* and *Actinobacilus pleuropneumoniae.* Therefore the most detailed scenarios will give the most useful and accurate information.

### Feasibility

Of course the most detailed scenarios may not be easily implemented at slaughter due to time-constraints and/or lack of human resources. Moreover, the reliability of the information gathered depends on the reproducibility of the scoring system, therefore scoring systems that entail some degree of subjectivity (e.g., differentiating between < 10% of affected lung surface and 10–30%) should be avoided.

Regarding the evaluation of CVPC, scenarios 3 and 4 only require OVs/AVs to identify these lesions and to count the different lung lobes affected. This information could be gathered with “only one click” and then translated into both the prevalence and severity of CVPC. In scenario 4, the exclusion of the intermediate lobe did not lead to a loss of information when compared to scenario 3 (Fig. 1). This exclusion would allow OVs/OAs to save time by not having to rotate the lungs to examine the intermediate lobe. Furthermore, with the implementation of visual only MI in the EU, scoring systems that entail palpation/rotation of the lungs should be discouraged. Based on these, we recommend the implementation of scenario 4 for the evaluation of CVPC during routine MI.

Regarding pleurisy, the most feasible scoring system is scenario 4 whereby only lungs retained in the carcass are registered. Unfortunately, this represents a substantial loss of information and the introduction of inaccuracies. As Scenario 3 does not involve scoring mild DC pleurisy cases, it reduces the OVs/OAs workload while still allowing for the differentiation between CP and DC. It also provides a measure of the severity of DC pleurisy lesions, which is not attainable by scenarios 1 and 2. Therefore, we recommend the implementation of scenario 3 for the evaluation of pleurisy lesions during routine MI.

Clearly, the proposed scenarios should be tested and validated not only by the OVs/OAs implementing the scoring systems, but also by private veterinarians and farmers who will use the information generated to sustain decision making on farm.

## Conclusion

The insights gained from this study are applicable to the current EU efforts to improve data collection at MI. The optimal simplified CVPC scoring system involves counting the number of lung lobes affected while excluding the intermediate lobe (scenario 4). This provides the best trade-off between value of information and feasibility by incorporating information on both the prevalence and severity of CPVC. Pleurisy is best evaluated by considering the presence or absence of cranial pleurisy while scoring only moderate and severe lesions in the dorsocaudal region (scenario 3). However, further validation at slaughter and by private veterinarians and farmers is needed for both scenarios.

## Data Availability

The datasets used and/or analyzed during the current study are available from the corresponding author on reasonable request.

## Abbreviations

CP: Cranial pleurisy
CVPC: Cranioventral pulmonary consolidation
DC: Dorsocaudal pleurisy
EU: European Union
MI: Meat Inspection
OAs: Official auxiliaries
OVs: Official veterinarians
SPES: Slaughterhouse Pleurisy Evaluation System
